# Attention deficit hyperactivity disorder and future alcohol outcomes: Examining the roles of coping and enhancement drinking motives among young men

**DOI:** 10.1371/journal.pone.0218469

**Published:** 2019-06-19

**Authors:** Véronique S. Grazioli, Gerhard Gmel, Ansgar Rougemont-Bücking, Stéphanie Baggio, Jean-Bernard Daeppen, Joseph Studer

**Affiliations:** 1 Addiction Medicine, Lausanne University Hospital CHUV, Lausanne, Switzerland; 2 Addiction Switzerland, Lausanne, Switzerland; 3 Centre for Addiction and Mental Health, Toronto, Canada; 4 University of the West of England, Bristol, United Kingdom; 5 Division of Prison Health, Geneva University Hospitals, Geneva, Switzerland; 6 Life Course and Social Inequality Research Centre, University of Lausanne, Lausanne, Switzerland; University of Oviedo, SPAIN

## Abstract

**Objective:**

Although there is evidence that Attention Deficit Hyperactivity Disorder (ADHD) symptoms are positively related to alcohol use and related problems among young adults, little research has examined the mechanisms that might explain this association. In response, this study examined the mediating effects of coping and enhancement drinking motives on the prospective associations between ADHD symptoms and alcohol outcomes.

**Method:**

Participants (*N* = 4,536) were young men from the Cohort Study on Substance Use Risk Factors. Measures of ADHD symptoms and those of drinking motives, heavy episodic drinking (HED) and alcohol use disorder symptoms were used from the baseline and 15-month follow-up assessments.

**Results:**

Findings indicated that the associations of ADHD-inattention symptoms with alcohol use disorder (AUD) symptoms and with HED were partially and completely mediated through drinking motives, respectively, whereas drinking motives did not mediate the ADHD-hyperactivity/impulsivity-symptoms-alcohol outcomes associations.

**Conclusion:**

Results indicated that coping and enhancement motives partially explained the ADHD-inattention symptoms—subsequent alcohol outcomes association. These findings suggest that interventions targeting enhancement and coping motives may help prevent problematic drinking among young men with elevated ADHD-inattention symptoms.

## Introduction

Attention Deficit Hyperactivity Disorder (ADHD) is a neurodevelopmental disorder characterized by inattentive (e.g., often distracted by extraneous stimuli) and/or hyperactive-impulsive symptoms (e.g., excessive talking) affecting 5–10% of school-age children [1, 2]. Contrary to the widespread belief that ADHD outgrows as children mature, longitudinal studies have shown that ADHD symptoms persist into adulthood in up to 65% of cases [3]. Accordingly, epidemiological studies indicate that adult ADHD prevalence is between 1.0–7.5% worldwide, [4–8], with prevalence rates being higher in young males (5.4%) than in young females (3.2%) [4]. Alarmingly, adult ADHD has been consistently related to social (e.g., high prevalence of adult ADHD in incarcerated populations) and professional functional impairments (e.g., unemployment), thereby producing costly societal burden [4, 9–12]. Furthermore, psychological comorbidities (e.g., depression, antisocial personality disorder) are common among adults with ADHD, potentially leading to further functional impairments [5, 8, 13].

There is also evidence that ADHD is a correlate of problematic drinking in adults [14, 15]. Notably, besides often co-occurring, there is evidence that ADHD and alcohol use disorder (AUD) share similar signs and symptoms [16]. Specifically, both ADHD and AUD have been associated with neuropsychological impairments in diverse domains, including executive functioning and working memory [1, 17, 18]. Given their similarities and their co-occurrence, research has started to examine both ADHD and AUD together. A line of this research has focused on the mechanisms through which ADHD may promote alcohol outcomes. This study aims to contribute to this field of research by examining whether coping and enhancement drinking motives—drinking to attenuate negative affects and to enhance positive mood or well-being, respectively—mediate the prospective association between ADHD symptoms and alcohol outcomes among young men. Examining this question may be especially relevant in young males considering their elevated rates of both ADHD prevalence and risky drinking behaviors. Indeed, as mentioned earlier, prevalence rate of ADHD is higher among young than among young females [4]. Furthermore, among young adults, males have been consistently identified as being at greater risk regarding unhealthy drinking behaviors than females [19, 20]. Epidemiological research indicates for instance that prevalence rates of heavy episode drinking (HED, reporting > 60gr of pure alcohol on a single occasion) in the past 30 days are twice as high among young males compared with young females (i.e., ages 15–24; [21]).

Increasing evidence suggests that ADHD stands at the end of a continuum and that ADHD symptoms may occur in the absence of the full disorder [22, 23]. Accordingly, recent research has started to examine manifestations of ADHD symptoms even if they do not meet diagnostic threshold (e.g., [13, 24, 25]). Such examination allows capturing subclinical variations along the ADHD continuum, thereby providing valuable information for prevention. There is evidence that ADHD symptomatology is positively related to alcohol outcomes in nonclinical samples of youths and adults [14], even though some findings have been inconsistent across studies regarding inattention and hyperactivity/impulsivity dimensions [26, 27]. For instance, studies in youths (i.e., adolescents) found ADHD symptoms to be positively related to drinking frequency [14]. Hyperactivity/impulsivity (hereafter referred as ADHD-HI) symptoms have been positively related to alcohol use and related problems in adults and college students [13, 27], yet they were not significantly related to alcohol use and related-problems in two other studies among college students [24, 26]. Finally, some previous findings in the same populations have documented inattention symptoms (hereafter referred as ADHD-I) to be positively associated with alcohol use [24, 27], whereas others found opposite results [26]. Similarly, ADHD-I symptoms were found to be associated with alcohol-related problems in one research [27], whereas another study found this association to be significant among females, yet not among males [13].

Recent research has started to examine the mechanisms through which ADHD may promote alcohol outcomes. That said most studies examining this line of research has focused on the associations between childhood ADHD and subsequent AUD. For instance, a study conducted in the general adult population found that the positive association between childhood ADHD and future AUD was mediated by conduct disorders [28]. In another study, moderated-mediation analyses indicated that social impairment, delinquency and grade points significantly mediated the association between childhood ADHD and subsequent alcohol use among youths with lower parental knowledge of their child friendships, activities and whereabouts [29]. Some of these findings were corroborated by another study that found social impairment and delinquency as significant mediators of the association between childhood ADHD and future HED [30].

To the best of the authors’ knowledge, only three studies to date have attempted to identify factors that may explain the associations between ADHD symptoms and alcohol outcomes in samples of non-clinical adults. The two first studies were conducted among college students and identified disinhibition, difficulty stopping drinking, lack of premeditation and sensation seeking as significant mediators of the positive association between ADHD (diagnoses, symptoms) and current alcohol use and/or related consequences [27, 31]. The third study was conducted among college students in China [32]. The authors found that anxiety significantly mediated the association between ADHD-HI symptoms and alcohol behaviors (i.e., alcohol use, HED, intoxication), whereas depression mediated both the associations between ADHD-HI and alcohol behaviors, and the one between ADHD-IN and alcohol behaviors.

Other correlates of drinking that may play a role in the ADHD symptoms-alcohol outcomes association include drinking motives, which are considered among the most proximal factors for engaging in drinking behaviors [33, 34]. According to the motivational model of alcohol use, some individuals drink alcohol to enhance positive mood or well-being (i.e., enhancement drinking motives), and others to attenuate negative affects (i.e., coping drinking motives; [33, 35]). Given that ADHD is related to functional impairments and psychiatric problems, it is likely that individuals with elevated symptoms are motivated to use alcohol to seek relief from psychological distress and thus to endorse high coping drinking motives. In fact, coping with stressful events may be particularly challenging for individuals with ADHD because of executive functioning impairments, including inattention and distractibility [36, 37]; specifically, attention deficits may impede them to cognitively reappraising stressful situations and using adaptive strategies. Accordingly, a study conducted among adults with ADHD showed maladaptive coping strategies (e.g., escape-avoidance, less problem solving) to be the most endorsed in this population [38]

Similarly, it is likely that individuals with elevated ADHD symptoms endorse high enhancement drinking motives; indeed, considering that two core ADHD symptoms include being easily bored and having aversion of delayed reward, alcohol use may represent a means of reducing boredom feelings by providing immediate stimulation [39]. Echoing this hypothesis, past research has found significantly higher levels of sensation seeking (i.e., tendency to seek stimulation, novelty and risk; [40]) among individuals with ADHD than among controls [41, 42], with sensation seeking being mostly related to the hyperactivity/impulsivity dimension rather than to the inattention one [43].

As far as we are aware of, the mediating effect of coping and enhancement drinking motives on the association between ADHD symptoms and alcohol outcomes in adults has not been tested yet. As outlined below, very little research to date has explored mediators of the association between ADHD symptoms and alcohol outcomes in adults. Answering a call to conduct more research examining potential explanatory factors of this association in large samples [26], this study aimed at examining the mediating effect of enhancement and coping drinking motives on the ADHD symptoms-alcohol outcomes association in a large sample of young men in Switzerland. Gaining a better knowledge of the mechanisms explaining the association between ADHD symptoms and alcohol outcomes is important to tailor interventions aiming to decrease problematic drinking to young adults with ADHD symptoms. Specifically, this study aimed at evaluating the mediating effect of coping and enhancement drinking motives on the prospective associations of ADHD symptoms (ADHD-total symptoms [ADHD-I and ADHD-HI symptoms]; ADHD-I symptoms and ADHD-HI symptoms separately) with HED and AUD symptoms in a representative sample of young men in Switzerland.

Based on previous research, we expected that ADHD symptoms would be positively related to subsequent alcohol outcomes. We also expected that these associations would be partially mediated by both drinking motives with models testing ADHD-HI symptoms being mediated by enhancement drinking motives and those testing ADHD-I by coping drinking motives.

## Methods

### Procedure and participants

This study used data from the Cohort Study on Substance Use Risk Factors designed to examine substance-use trajectories among young Swiss men. Recruitment took place between August 2010 and July 2011 in three of a total of six recruitment centers covering 21 of the 26 Swiss cantons (including French and German-speaking cantons). In Switzerland, all Swiss men aged approximately 19 must undergo a recruitment process to determine their eligibility for military service. All young men were eligible for inclusion, independently of whether they were deemed eligible or not to serve in the army. Thus, virtually all men aged 19–20 in the 21 covered cantons were eligible for inclusion in the study. Participants completed questionnaires outside of the army environment. At baseline, conscripts who gave consent to participate were invited by email (or by mail if they asked so) within two weeks to complete a paper pencil questionnaire or an online questionnaire. Then, they received an email (or a mail if they preferred) to complete the follow-up assessment using an online questionnaire (or a paper-pencil questionnaire if they preferred; 45–60 minutes duration on average). Participants received a voucher after completion of each survey (i.e., CHF 30—around $30 for the baseline and for the follow-up 1 and CHF 50—around $50 for the follow-up 2 assessment). More details regarding the parent study procedures are provided elsewhere [44]. In the current study, we used data from the baseline and 15-month follow-up assessments. All procedures were approved by the institutional review board (ethic committee of the Canton de Vaud, research protocol number 15/07).

**Fig 1:**
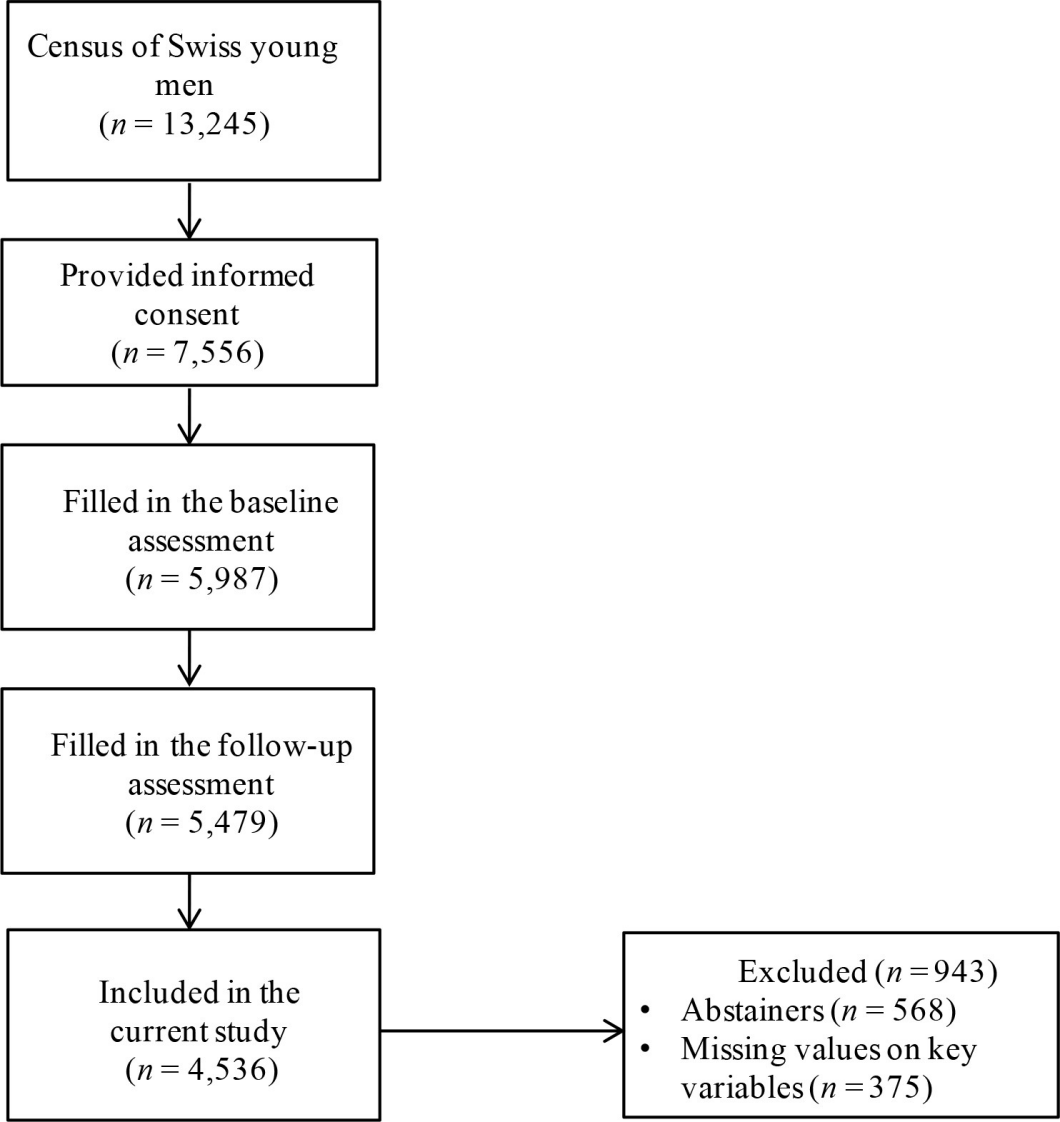
Flow diagram documenting participants’ progression from recruitment to participation in the current study.

A total of 7,556 conscripts provided written informed consent to participate in the study. Of those, 5,987 (79.2%) completed the baseline assessment between September 2010 and March 2012, and among them 5,479 (91.5% of the baseline sample) completed the follow-up assessment between March 2012 and January 2014. Non-response analysis indicated that non-respondents reported more alcohol use than respondents. The magnitude of these differences was, however, small, indicating a small non-response bias [45]. For instance, non-respondents (9.5%) were more often abstainers than respondents (9.2%) but the difference was not significant (OR = 1.04, 95% CI 0.91, 1.17), whereas non-respondents reported HED at least monthly significantly more often than respondents (49.4% vs 45.1%, OR = 1.19, 95% CI 1.10, 1.28). Abstainers (*n* = 568, 10.4%) were not included in the current study because the questionnaire assessing drinking motives was administrated among 12-month drinkers only. Missing values (*n* = 375, 6.8%) on key variables were listwise deleted, resulting in a final sample of 4,536 participants (see Fig 1). At baseline, the mean age of participants was 19.94 (*SD* = 1.19). More than half of the sample was French-speaking (54.30%). Primary school was the most commonly reported highest level of education completed (48.80%), followed by secondary school (28.30%) and tertiary school (22.80%).

### Measures

#### Socio-demographic variables

Age, linguistic region (i.e., German and French-speaking) and highest achieved education were assessed at baseline and served to describe the sample and as covariates in the analyses.

#### Adult ADHD symptoms

Adult ADHD symptoms over the past 12 months were assessed at baseline with the Adult ADHD Self-Report Scale Screener (ASRS-v1; [46]). The ASRS-v1.1 is a six-item scale that measures six ADHD symptoms based on DSM-IV diagnostic criteria for ADHD [47]. Participants indicated how often they experienced each symptom over the past 12 months with a Likert-scale ranging from 0 to 4. The ASRS-v1.1 includes a 4-item inattention subscale (e.g., *How often do you have problems remembering appointments or obligations*) and a 2-item hyperactivity/impulsivity subscale (e.g., *How often do you feel overly active and compelled to do things*, *like you were driven by a motor;* [48]). ADHD-total symptoms (α = 0.80) and ADHD-I symptoms (α = 0.80) showed adequate internal consistency, unlike ADHD-HI symptoms (α = 0.66), which was not unexpected given that this subscale comprised only two items. ADHD symptoms were treated as latent variables for ordinal data in the main analyses. Specifically, each item was constrained to load on its respective factor. These measures served as independent variables in the analyses.

#### Drinking motives

Coping and enhancement drinking motives were measured at 15 months with two 3-item subscales of the Drinking Motives Questionnaire Revised Short Form (DMQ-R SF; [49]). Participants were asked to consider all the time they had consumed alcohol in the past year and indicate how often they did so for coping (e.g., *to forget about your problems*) or enhancement (e.g., *to get high*) motives, using a 5-point Likert scale. Coping (α = 0.84) and enhancement (α = 0.82) drinking motives subscales showed adequate consistency. Both drinking motives were treated as latent variables for ordinal data in the analyses and served as mediators in the analyses.

#### Alcohol outcomes

AUD symptoms following DSM-5 diagnostic criteria were measured with 11 items adapted from the Semi-Structured Assessment for the Genetics of Alcoholism (SAAGA; [50, 51]). Participants were asked to indicate whether they had experienced any of 11 situations corresponding to AUD symptoms over the past 12 months (e.g., *you often found yourself drinking more and for longer periods of time than you intended*). Reliability and validity of this measure have been supported in previous research [50, 51]. In this study, internal consistency was adequate (α = 0.70). AUD symptoms were treated as a latent variable for ordinal data in the analyses. Participants were also asked to indicate how often they drank six or more alcoholic beverages (≥ 60gr of pure alcohol) on one occasion in the past 12 months with a Likert scale ranging from 0 to 5. Answers were dichotomized to yield a report of monthly HED, where 0 = *reporting less than 1 HED per month* and, 1 = *reporting one or more HED per month*. AUD symptoms and HED at 15 months and baseline served as dependent variables and covariates, respectively.

#### Depression symptoms

Depression symptoms were assessed at baseline with the Major Depression Inventory (MDI; [52]), which is a 10-item scale covering the ICD-10 symptoms of depression such as *feeling lacking in energy and strength* [53]. Participants were asked to indicate how often they had experienced each symptom over the past 2 weeks using a 6-point Likert scale ranging from 0 to 5. Answers were summed up to yield a total score (depression symptoms), which served as covariate in the analyses. Internal consistency was good (α = 0.89).

#### Anti-social personality disorder symptoms

The Mini International Neuropsychiatric interview (MINI plus; [54]) was used to assess anti-social personality disorder (ASPD) symptoms. Participants were required to indicate how often they had engaged in 12 behaviors—six before they were 15 years old (e.g., *Before you were 15 years old*, *how often did you start fights or bully*, *threaten*, *or intimidate others*?) and in six others since they were 15 (e.g., *Since you were 15 years old*, *how often have you exposed others to danger without caring*?)—using a 6-point Likert-scale ranging from 1 to 6. Answers were summed up to yield a total score (ASPD symptoms), which served as covariate in the analyses. Internal consistency was adequate (α = 0.83).

### Statistical analyses

**Fig 2:**
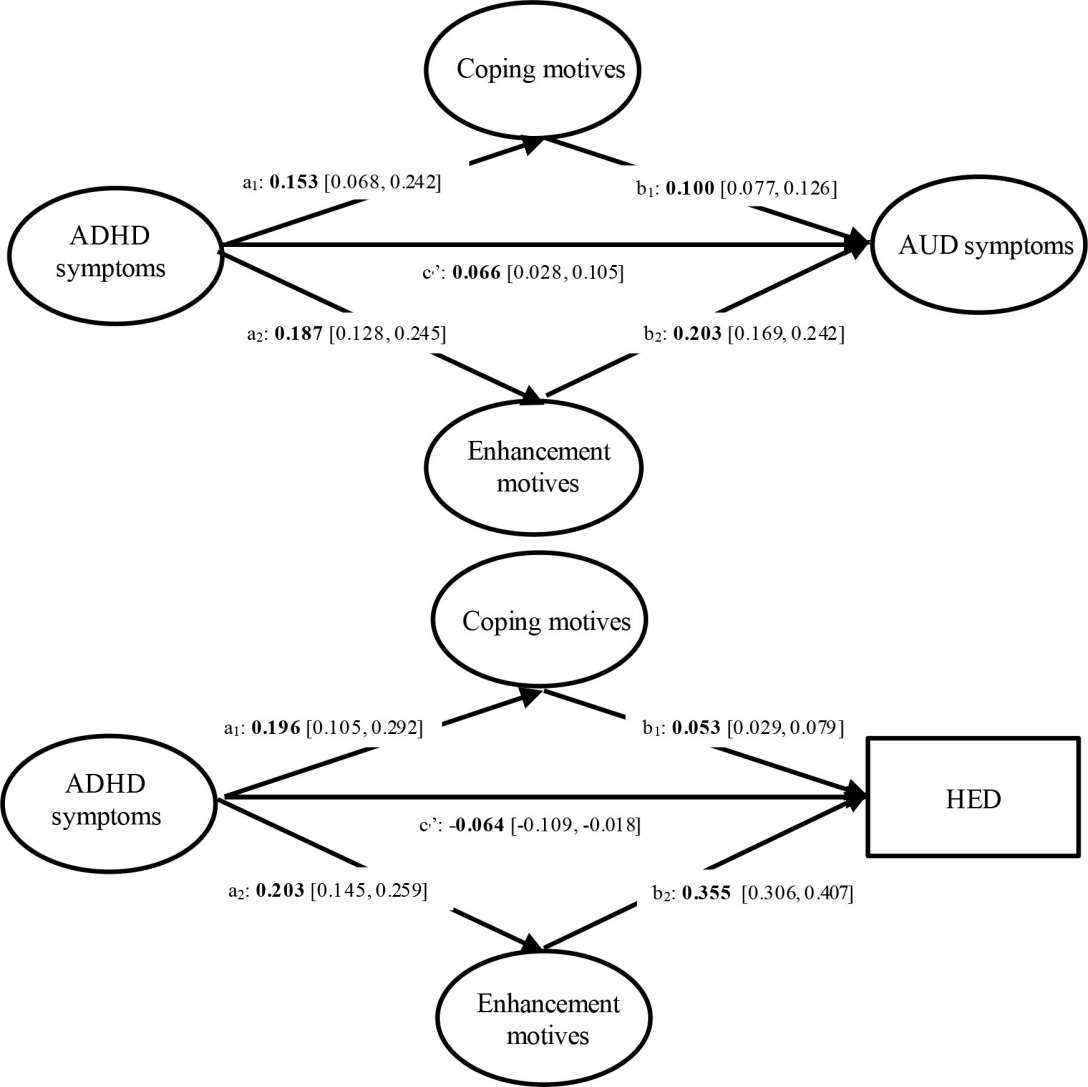
Results of the structural equation model of ADHD-total symptoms. Covariates were omitted from the figure for ease of presentation. For ease of reading, significant parameters are shown in bold.

Four structural equation models (SEMs) were conducted to examine the path from ADHD symptoms to alcohol outcomes through coping and enhancement drinking motives. Two SEMs examined the mediating effects of drinking motives on the associations between ADHD-total symptoms at baseline and a) AUD symptoms and b) HED at 15 months; two other SEMs tested the mediating effects of drinking motives on the associations of ADHD-I and ADHD-HI symptoms at baseline with c) AUD symptoms and d) HED at 15 months. To test mediation (see Fig 2 for illustrative purposes), SEMs estimate the path (*c’*) between the predictor (e.g., ADHD symptoms) and the outcome (i.e., alcohol outcomes), the paths (*a*) between the predictor and the mediators (e.g., coping and enhancement drinking motives) and the paths (*b*) between the mediators and the outcome. Specific indirect associations of the predictor on the outcome through a specific mediator is the product of the paths linking the predictor to the given mediator (*a*) and the one linking that moderator to the outcome *b*); the total indirect associations of a predictor is the sum of all specific indirect associations, whereas the total association is the sum of the direct association (*c’*) and the total indirect association. SEMs show partial mediation when both indirect and direct associations are significant and full mediation when the indirect association is significant, whereas the direct association is not.

All models were adjusted for demographic variables. Furthermore, given that comorbidities with depression and ASPD are common in adult ADHD [8, 55, 56] and that they are related to problematic alcohol use [57–59], all models were adjusted for depression and ASPD symptoms at baseline. Finally, the AUD and HED SEMs were adjusted for AUD and HED at baseline, respectively. Parameter estimates were based on the weighted least squares mean-variance adjusted estimator, which was developed to handle ordinal indicators [60]. We tested for mediation using the 95% bias-corrected bootstrap confidence intervals based on 5,000 bootstrap samples, to account for potential nonnormality of the indirect effect [61, 62]. All analyses were conducted with and without adjustment. Model fit was examined using the comparative fit index (CFI) and the root mean square error of approximation (RMSEA). A CFI higher than 0.95 and a RMSEA close to 0.06 or lower indicate a good fit [63]. Descriptive analyses and the SEMs were conducted on SPSS 23 and on Mplus 7, respectively.

## Results

Descriptive statistics and correlations among key variables are presented in Table 1. Fit indices indicated that the SEMs achieved a good fit (all CFIs > 0.95; all RMSEAs < 0.05). Unadjusted models showed the same general pattern than adjusted ones. We thus present below results yielded in the adjusted models only.

**Table 1:** Descriptive statistics and bivariate correlations among key variables (N = 4,536).

Variable					Correlations^a^						
	M/%	SD	Skewness	Kurtosis	1	2	3	4	5	6	7
**ADHD symptoms baseline**											
1. ADHD-total	5.69	4.24	0.47	-0.25	-						
2. ADHD-I	3.39	2.94	0.81	0.42	0.91***	-					
3. ADHD-HI	2.29	1.97	0.56	-0.42	0.82***	0.53***	-				
**Drinking motives 15-months**											
4. Coping motives	1.63	0.78	1.28	1.08	0.13***	0.14***	0.08***	-			
5. Enhancement motives	2.63	1.10	0.05	-0.99	0.19***	0.20***	0.11***	0.37***	-		
**Alcohol outcomes 15 months**										
6. HED at least 1/month	51.8%				0.07***	0.07***	0.04***	0.27***	0.46***	-	
7. AUD symptoms	1.32	1.64	1.72	3.87	0.21***	0.22***	0.15***	0.35***	0.46***	0.44***	-

^a^Spearman rank-order correlations. ADHD-total, ADHD-I and ADHD-hyperactivity symptoms scores ranged from 0 to 24, 0 to 16 and 0 to 8, respectively. Coping and enhancement drinking motives scores ranged from 1 to 5. AUD symptoms scores ranged from 0 to 11.

*** *p* < .001.

### Coping and enhancement drinking motives as mediators of the association between ADHD-total symptoms and alcohol outcomes

#### AUD symptoms

As shown in Table 2 and Fig 2, the total and direct (c_1_’ on Fig 2) associations between ADHD-total symptoms and AUD symptoms and the specific indirect associations through coping and enhancement motives were significant. These findings indicated that the positive association between ADHD-total symptoms and AUD symptoms was partially mediated through coping and enhancement motives, such that ADHD-total symptoms at baseline were positively related to drinking motives at 15 months, which in turn were positively related to AUD symptoms.

**Table 2:** Structural equation models examining the mediating effects of coping and enhancement drinking motives on the association between ADHD-total, I and HI symptoms and AUD symptoms and HED (N = 4,536).

	AUD symptoms			HED		
	*B*	SE	[95%CI]	*B*	SE	[95%CI]
**ADHD-total symptoms models**^1^						
** Total association**	**0.119**	0.021	[0.078, 0.163]	0.019	0.024	[-0.028, 0.067]
** Total indirect association**	**0.053**	0.009	[0.036, 0.071]	**0.082**	0.012	[0.059, 0.106]
** Specific indirect associations**						
Through coping (a_1_ x b_1)_	**0.015**	0.005	[0.007, 0.025]	**0.010**	0.004	[0.005, 0.019]
Through enhancement (a_2_ x b_2_)	**0.038**	0.007	[0.026, 0.052]	**0.072**	0.011	[0.050, 0.094]
**ADHD-I and -HI symptoms models**^2^ **ADHD-I symptoms**						
** Total association**	**0.134**	0.030	[0.078, 0.197]	0.037	0.037	[-0.033, 0.111]
** Total indirect association**	**0.073**	0.014	[0.048, 0.101]	**0.103**	0.018	[0.069, 0.140]
** Specific indirect associations**						
Through coping (a_1_ x b_1_)	**0.023**	0.007	[0.010, 0.039]	**0.015**	0.005	[0.006, 0.027]
Through enhancement (a_2_ x b_2_)	**0.050**	0.010	[0.033, 0.071]	**0.089**	0.016	[0.058, 0.122]
**ADHD-HI symptoms**^2^						
** Total association**	-0.013	0.020	[-0.053, 0.025]	-0.015	0.027	[-0.073, 0.035]
** Total indirect association**	-0.017	0.009	[-0.036, 0.001]	-0.017	0.012	[-0.042, 0.006]
**Specific indirect associations** Through coping (a_3_ x b_1_)	-0.007	0.005	[-0.018, 0.002]	-0.003	0.003	[-0.010, 0.001]
Through enhancement (a_4_ x b_2_)	-0.010	0.007	[-0.024, 0.002]	-0.014	0.011	[-0.036, 0.007]

*Note*. *B*, unstandardized slopes; SE, standard error of *B*. The confidence interval reflects the 95% bias-corrected bootstrap confidence interval based on 5,000 bootstrapped samples. The models were adjusted for demographic variables, depression, ASPD and alcohol outcome at baseline.

For ease of reading significant parameters are shown in bold.

^1^The letters refer to Fig 1.

^2^The letters refer to Fig 2.

#### HED

Findings showed a significant direct (c_1_’ on Fig 2) association, such that ADHD-total symptoms were negatively related to HED. The total association between ADHD-total symptoms and HED was not significant. However, it is possible to have significant mediation (indirect effects) in the absence of a total effect [64]. The indirect associations through both motives did reach significance, indicating that ADHD-total symptoms were positively associated with drinking motives at 15 months, which were in turn positively related to HED (see Table 2, Fig 2).

### Coping and enhancement drinking motives as mediators of the association between ADHD-I and ADHD-HI symptoms and alcohol outcomes

#### AUD symptoms

**Fig 3:**
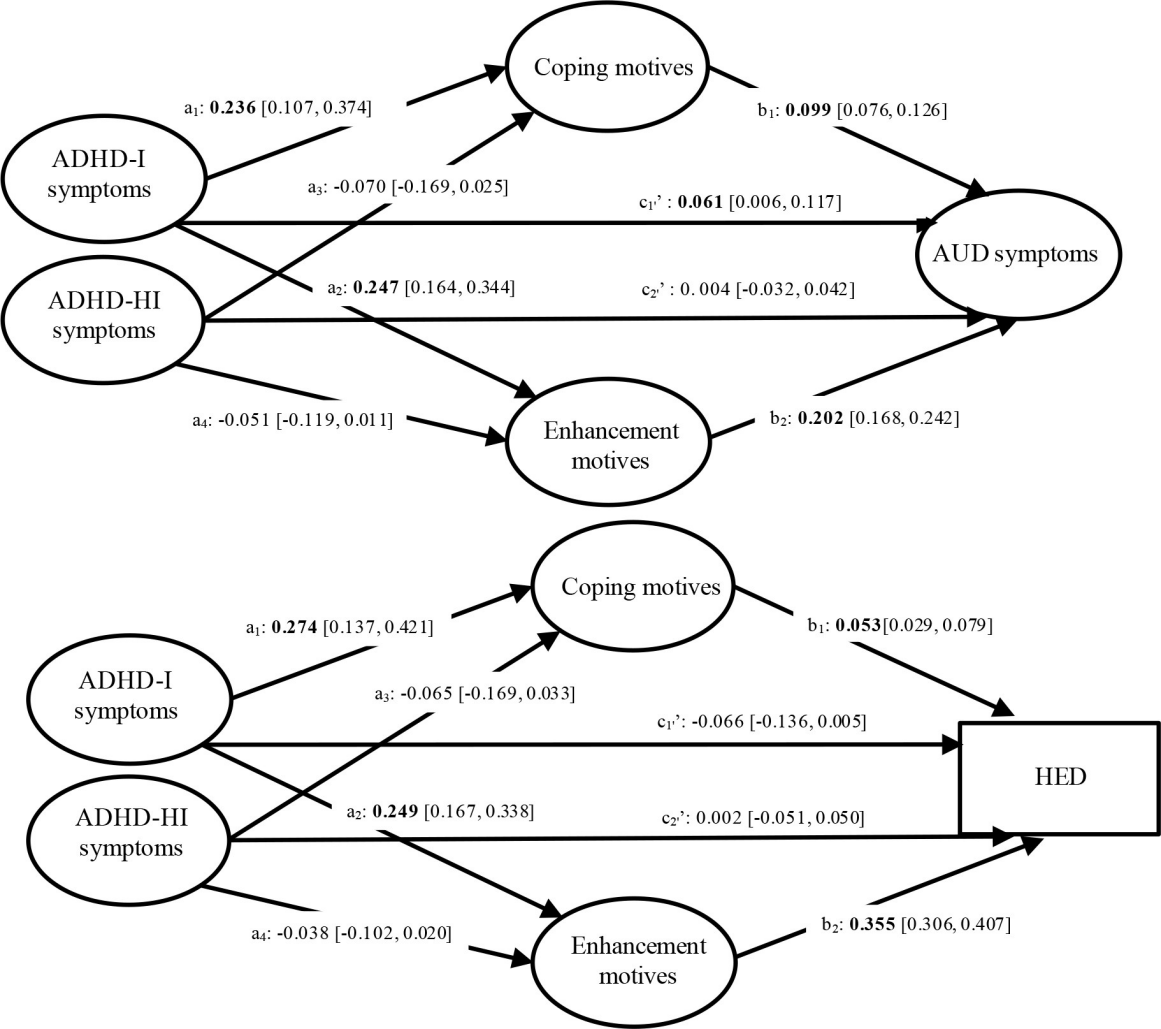
Results of the structural equation model of ADHD-I and HI symptoms. Covariates were omitted from the figure for ease of presentation. For ease of reading, significant parameters are shown in bold.

The total and direct (c_1_’ on Fig 3) associations between ADHD-I symptoms and AUD symptoms were significant, so were the specific indirect associations through coping and enhancement motives (see Table 2 and Fig 3). These findings revealed that the positive association between ADHD-I symptoms and AUD symptoms was partially mediated by drinking motives, such that ADHD-I symptoms at baseline were positively related to drinking motives at 15 months, which were in turn positively related to AUD symptoms. Regarding ADHD-HI-symptoms, the total and the direct (c_2_’ on Fig 3) associations between ADHD symptoms and AUD symptoms did not reach significance. Similarly, the specific indirect associations through drinking motives were not significant (see Table 2 and Fig 3).

#### HED

The total association between ADHD-I symptoms and HED was not significant, neither was the direct association (c_1_’ on Fig 3). Next, the specific indirect associations through coping and enhancement motives were significant, such that ADHD-I symptoms were positively associated with drinking motives at 15-months, which were in turn positively related to HED. Finally, neither the total association between ADHD-HI symptoms and HED, nor the direct (c_2_’ on Fig 3) and indirect associations through drinking motives did reach significance.

## Discussion

This study aimed to examine the mediating effects of coping and enhancement drinking motives on the prospective associations of ADHD-total, ADHD-I and ADHD-HI symptoms with HED and AUD symptoms among young males. As expected, findings indicated that enhancement and coping drinking motives significantly mediated the associations between ADHD-total symptoms and subsequent HED and AUD symptoms. Findings also revealed indirect effects of drinking motives on the association between ADHD-I symptoms and subsequent alcohol outcomes. By contrast, findings showed that neither enhancement nor coping drinking motives significantly mediated the association of ADHD-HI symptoms with subsequent alcohol outcomes.

Unexpectedly the SEMs yielded a significant negative direct association between ADHD-total symptoms and future HED. These findings are inconsistent with past research that revealed significant positive associations between ADHD symptoms and alcohol use beyond the effects of depression and antiestablishment attitudes [14]. Notably though, the latter study focused on adolescents, whereas the current study included young men. It is possible that the association between ADHD symptoms and alcohol use changes over time. In fact, another study conducted among college students found ADHD symptoms to be not significantly related to alcohol use [26], whereas another study documented that college students with ADHD reported lower rates of alcohol use than their non-ADHD peers [65]. Our findings that ADHD-total symptoms were negatively related to HED may also pertain to an inconsistent mediation representing the variance left over after taking coping and enhancement drinking motives into account. Accordingly, bivariate analyses documented significant positive associations between ADHD symptoms and subsequent HED, although coefficients were small. Given the significant indirect associations through coping and enhancement drinking motives, these findings may indicate that young adults with elevated ADHD symptoms are not likely to engage in HED if they are not seeking coping and enhancement from this behavior. Furthermore, in line with past research [15], findings indicated that ADHD symptoms were positively associated with subsequent AUD symptoms. Taken together, these findings suggest that endorsing ADHD symptoms may increase risks of engaging in problematic drinking in youths (i.e., endorsing AUD symptoms); young adults with elevated ADHD symptoms may therefore benefit from alcohol-related harm reduction interventions (e.g., personalized normative feedbacks, protective behavior strategies use promotion; [66, 67]).

Interestingly, findings did not support a significant association between ADHD-HI symptoms and alcohol outcomes (i.e., HED; AUD symptoms), whereas ADHD-I symptoms were significantly related to more AUD symptoms but not to HED. These results are incongruent with some of previous research conducted in young adults that revealed ADHD-HI symptoms to be significantly related to alcohol use [27, 32] and problematic alcohol use [13, 32], and ADHD-I symptoms associated with alcohol use [27, 32]. Notably though, both Tong and colleagues and Roberts and colleagues focused on ADHD-HI diagnostic thresholds rather than ADHD-HI symptoms, which may help explain these differences. That said our findings are consistent with another study conducted among college students that found ADHD-HI symptoms to be unrelated to alcohol use and related problems and ADHD-I symptoms to be significantly associated with alcohol-related problems, yet not alcohol use [26]. Our findings may pertain to the fact that ADHD-HI symptoms were measured with a 2-item subscale [48], although the ASRS-v1.1 has been found to have good psychometric properties [46, 68] and Glass and colleagues found similar findings while using a scale measuring ADHD-HI that included more items. It may also be that ADHD-I symptoms carry more importance than ADHD-HI symptoms among young adults. In fact, literature indicates that ADHD-I symptoms are more persistent than ADHD-HI symptoms over time [69, 70]. Importantly, given the inconsistent findings yielded in both past research and the current study, future research is needed to clarify the associations between ADHD-HI and ADHD-I symptoms and alcohol outcomes among young adults.

Our findings notably add to the existing literature by providing initial evidence that the association between ADHD symptoms and subsequent alcohol outcomes may be partially explained by coping and enhancement drinking motives. As expected, findings revealed indirect effects of enhancement and coping drinking motives on the associations between ADHD-total symptoms and subsequent HED and AUD symptoms. Further, findings revealed indirect effects through coping drinking motives on the association between ADHD-I symptoms and subsequent alcohol outcomes. Unexpectedly, findings also revealed significant indirect effects through enhancement drinking motives on the longitudinal association between ADHD-I symptoms and alcohol outcomes. It may be that impairments of executive functioning, such as inattention and distractibility, lead to poor risk perception and impaired ability to deploy appropriate judgment, which may in turn increase the likelihood of engaging in risky behaviors aiming to seek sensation (e.g., fast driving). Past research has documented that adults with ADHD are prone to risky decision making [71] and, relatedly, to engage in risky behaviors. In fact, a recent review of the literature on ADHD and driving risks concluded that deficits in cognitive abilities such as inattentiveness may be the mechanism through which ADHD affects driving risks [72].

Taken together, the findings of this study suggest that young adults with elevated ADHD-I symptoms may be more at risk from engaging in problematic drinking because they use alcohol for enhancement and/or coping motives. If replicated by future research, these findings provide important insight regarding the development of alcohol-related interventions tailored to young men endorsing elevated ADHD-I symptoms. Specifically, they suggest that alcohol-related cognitive behavioral interventions targeting both enhancement and coping drinking motives may be a promising way to prevent problematic drinking among young adults with elevated ADHD-I symptoms [73]. Such interventions might aim to increase awareness of coping and enhancement drinking motives as well as to reduce alcohol use as a coping mechanism by means of a coping skill training program (e.g., relaxation methods). Furthermore, they might target enhancement drinking motives by helping at-risk young men consider alternative ways to enhance their well-being and positive mood (e.g., sport practicing, mind-fullness meditating; [73]).

Although the major strengths of this study include its longitudinal design and its large sample size, findings should be interpreted in the light of several limitations. First, the sample was limited to young males, which precludes generalizability of findings to young females or to other age groups. Second, the study relied on responses to self-reported questionnaires and their validity may be a concern, although participants were assured confidentiality. Third, as mentioned earlier, ADHD-HI symptoms were measured with a 2-item subscale. Although the ASRS-v1.1 has been found to have good psychometric properties [46, 68], future research using additional scales is needed to further confirm these findings. Fourth, the association between mediators and outcome variables were measured at the same time point, which precludes drawing inferences regarding the temporal associations between these variables. Future research using additional follow-up measurements is thus necessary. Fifth, it is important to highlight that coefficients of the indirect effect through coping and enhancement drinking motives on the association between ADHD-total/ADHD-I and alcohol outcomes were rather small, suggesting small effect sizes. It may be that our models were inclusive of a wide range of measures, which may have diluted the overall effect sizes. That said, our results are supported by the fact that these indirect effects were consistently significant across both models. Future research further testing these indirect effects are yet needed to confirm these findings.

Despite these limitations, this study makes an interesting contribution to the literature by providing initial evidence that the association between ADHD-I symptoms and future alcohol outcomes is partially mediated by coping and enhancement drinking motives. Although future research is needed to confirm these findings and ensure their generalizability, they suggest that cognitive-behavioral interventions targeting enhancement and coping drinking motives may represent a promising way to decrease alcohol-related harm among at-risk young adults with elevated ADHD-I symptoms.
